# Systematic Construction and Validation of an Immune-Related Gene-Based Model to Predict Prognosis for Ovarian Cancer

**DOI:** 10.1155/2022/7356992

**Published:** 2022-04-21

**Authors:** Yihan Fu, Hong Sun

**Affiliations:** ^1^Obstetrics and Gynecology Hospital of Fudan University, 200082 Shanghai, China; ^2^Shanghai Key Laboratory of Female Reproductive Endocrine-Related Diseases, Shanghai, China 200082

## Abstract

Ovarian cancer (OC) is a malignancy with poor prognosis, stubborn resistance, and frequent recurrence. Recently, it has been widely recognized that immune-related genes (IRGs) have demonstrated their indispensable importance in the occurrence and progression of OC. Given this, this study aimed to identify IRGs with predictive value and build a prognostic model for a more accurate assessment. First, we obtained transcriptome and clinical information of ovarian samples from both TCGA and GTEx databases. After integration, we figured out 10 genes as immune-related prognostic genes (IRPGs) by performing the univariate Cox regression analysis. Subsequently, we established a TF-associated network to investigate its internal mechanism. The prognosis model consisting of 5 IRPGs was constructed later by lasso regression analysis. The comparison of the score with the clinical factors validated its independence and superiority in OC's prognosis. Moreover, the association between the signature and immune cell infiltration demonstrated its ability to image the immune situation of the tumor microenvironment. Finally, the reliability of the risk model was confirmed by the GEO cohort. Together, our study has constructed an independent prognostic model for OC, which may deepen the understanding of the immune microenvironment and help present novel biomarkers or ideas for targeted therapy.

## 1. Introduction

Ovarian cancer (OC), as one of the three malignancies that threatened females' heath over the world, has also been a hot spot in cancer research due to its highest mortality rate among all kinds of gynecological tumors. Since its hidden onset, 70% of women are in the advanced stage at diagnosis, resulting in poor prognosis [1]. Therefore, finding a more accurate early screening method is a priority for solving this dilemma.

The tumor microenvironment (TME) which incorporates immune cells, immune factors, and immune microenvironment is essential in the occurrence and development of malignancies [2]. Its effects include disrupting genomic stability, stimulating angiogenesis, anti-apoptosis, promoting cell proliferation, and shaping microenvironmental homeostasis [3]. Besides, it has been found that dysfunction of the immune system contributes to the destruction of immune surveillance [4]. As a malignant solid tumor, ovarian cancer possesses tumor cells, tumor-associated stromal cells, infiltrating immune cells, and other normal epithelial cells. Among them, emerging emphasis has been put on the importance of stromal cells in tumor progression and drug resistance. Meanwhile, the ability to infiltrate immune cells to participate in tumor invasion and metastasis has also attracted people's attention [5–8]. Several studies have proved the effects of targeting TME for tumor therapy [9]. Nevertheless, immunotherapies are still in the primary period, and our understanding of the value of IRGs for OC patients is still almost blank [10, 11]. Therefore, a cognitive understanding of OC's deeper genetic and immune properties is necessary to overcome this hurdle.

In this study, DEGs, which were compactly related to overall survival (OS), were determined by obtaining a large-scale sequencing database and using bioinformatics technology in the TCGA and GTEx databases. Then, combining the clinical information, we developed an immune-related gene-based signature. By validating it in the GEO dataset, we demonstrate the accuracy and stability of the model to help formulate therapeutic strategies for OC.

## 2. Materials and Methods

### 2.1. Data Preparation

RNA-Seq dataset of 88 normal ovarian tissues was obtained in the GTEx dataset (https://commonfund.nih.gov/GTEx). Transcriptome data of 379 ovarian cancer patients and clinical data were downloaded from TCGA (https://gdc.nci.nih.gov/). GSE49997 was adopted from the GEO database. The GSE49997 data were based on the GPL2986 platform and included 204 ovarian cancer patients. The preprocessing of data was as stated below: (1) The patients with insufficient clinical information were deleted (2) the patients whose overall survival (OS) <90 days were deleted (3) converted the unit of OS to day. As a result, we obtained 346 patients from the TCGA database as the training cohort for follow-up analysis and 178 patients from the GEO database as the validation cohort.

### 2.2. Identification of Differently Expressed Genes

We identified the DEGs between ovarian cancers and normal tissues by utilizing the R package limma. The thresholds were established as FDR <0.05 and |log2 foldchange| >2. After obtaining a series of identified immune-related genes (IRGs) in the ImmPort database (http://www.immport.org), the immune-related DEGs were screened out by conducting the correlation analysis (FDR<0.05 and |log2 foldchange| >1). The effects of immune-related DEGs were explored by the Gene Ontology (GO) and Kyoto Encyclopedia of Genes and Genomes (KEGG) analysis via the cluster profiler package in R. The difference of TF gene between the normal ovarian tissue and ovarian cancer tissue was determined by logFC >2, *P* < 0.05.

### 2.3. Construction of Risk Model

To further screen immune-related DEGs with prognosis value, the KM survival analysis was performed using the “survival” package, P <0.05 were considered as significance . Subsequently, LASSO regression analysis was carried out to construct a prognostic model through the “glmnet” package. To prevent overfitting, 1000 rounds of cross-validation were used to select the adjustment parameters.

### 2.4. Risk Score Calculation

The coefficient value for each gene was calculated based on the result of multivariate cox analysis. The patients' risk score is obtained from the following equation: Risk score = expression of gene1 × *β*gene 1 + expression of gene2 × *β*gene2 + ⋯+expression gene *x* × *β*gene *x*, where*x*is the number of genes and*β*is the coefficient value for each gene.

The patients in both TCGA and GEO cohorts were divided into high-risk and low-risk groups according to the median value.

### 2.5. Construction of the TF Network

To explore internal mechanisms among 10 IRPGs, a TF-associated network was established. We attained 318 transcription factors (TFs) from the Cistrome Cancer (http://cistrome.org/CistromeCancer/), which is regarded as a valuable website for biomedical and genetic-related study [12]. We picked up survival-related TFs by univariate cox analysis (corfilter >0.3, *P* < 0.05). Later, the Poisson correlation analysis was chosen to define the association between prognosis-related TF and 10 IRPGs (|correlation coefficient| >0.3 and *P* < 0.001); finally, we established the regulatory network using Cytoscape software (version 3.6.0) [13].

### 2.6. Survival Analysis

The Kaplan–Meier analysis was conducted to distinguish the survival rate among high and low-risk score groups via R's survival package. Besides, the receiver operating characteristic (ROC) curves determined by the area under the ROC curve (AUC) verified the accuracy and sensitivity of the prognosis prediction. AUC >0.60 was viewed as an acceptable predictive value, and AUC >0.75 was considered a good predictive value [14].

### 2.7. Statistical Analyses

The Wilcox test was applied to validate the coloration between risk score and different clinicopathological parameters. Univariate and multivariate Cox regressions were performed to determine whether the model could influence or be regarded as an independent biomarker of OC's clinical outcoming.

### 2.8. Investigation of Tumor-Infiltrating Immune Cell

The expression of 6 infiltrating immune cells (B cells, CD4+ T cells, CD8+ T cells, neutrophils, macrophages, and dendritic cells) in OC was obtained in the Tumor Immune Estimator Resource (TIMER) database (https://cistrome.shinyapps.io/timer/) [15]. Then, the “estimate” package was chosen to explore the association between prognostic signature with tumor-infiltrating immune cells. *P* < 0.001 was set as significance.

### 2.9. External Validation in the GEO Database

To testify the risk model's predictive value, GSE4997 from the GEO database was picked as the validation queue. Risk score calculation, groups dividing, KM, and ROC curve analyses were conducted as stated before.

### 2.10. Statistical Analysis

All statistical analyses were performed by the R software version 4.0.3. Maps were plotted using R-package “ggplot2.”

## 3. Results

### 3.1. Identification of IRGs in OC

**Figure 1:**
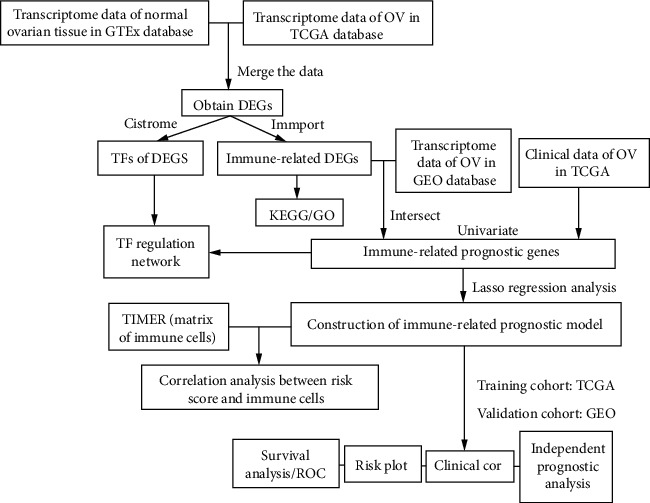
Flow diagram for establishing an immune-related prognostic model for OC.

**Figure 2:**
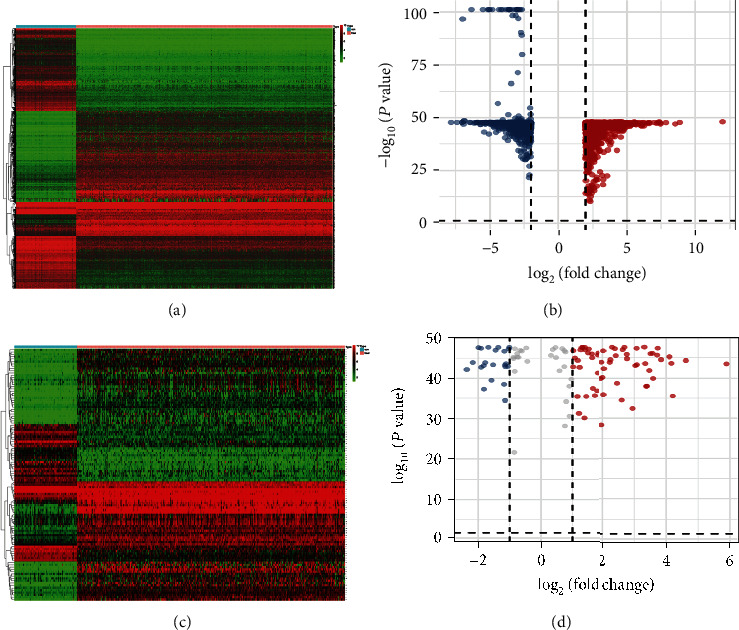
DEGs and immune-related DEGs in OC. A heatmap (a) and a volcano plot (b) of DEGs. A heatmap (c) and a volcano plot (d) of immune-related DEGs. The heatmap colored from green to red means the progression from low expression to high expression. The areas below the blue band represent normal samples, and the areas below the red band represent tumor samples. Colored dots in the volcano map on behalf of DEGs. Red stands for higher expression, blue stands for lower expression, while gray on behalf of the difference is not significant between normal and tumor samples.

The detailed workflow is presented in Figure 1. First, by acquiring and integrating datasets from TCGA and GTX database, 2253 DEGs that meet the set criteria (log FC| > 2, *P* *value* < 0.05) were filtered out in 379 OC tissues compared with 88 normal tissues, consisting of 1017 overexpressing and 1236 downexpressing genes, (Figures 2(a) and 2(b)).

Subsequently, we intersected these DEGs with immune-related genes detected in the ImmPort database to screen immune-related genes (IRGs); 181 IRGs were figured out in that way. Subsequently, in the same way, we obtained immune genes in GSE 49997 from GEO (|log FC| >1, *P* < 0.05), and 84 immune-related DEGs were screened after the intersection with the 181 IRGs for further analysis. As depicted in the heatmap (Figure 2(c)) and a volcano plot (Figure 2(d)).

### 3.2. Gene Ontology (GO) Term and Kyoto Encyclopedia Genes and Genomes (KEGG) Analyses for IRGs

**Figure 3:**
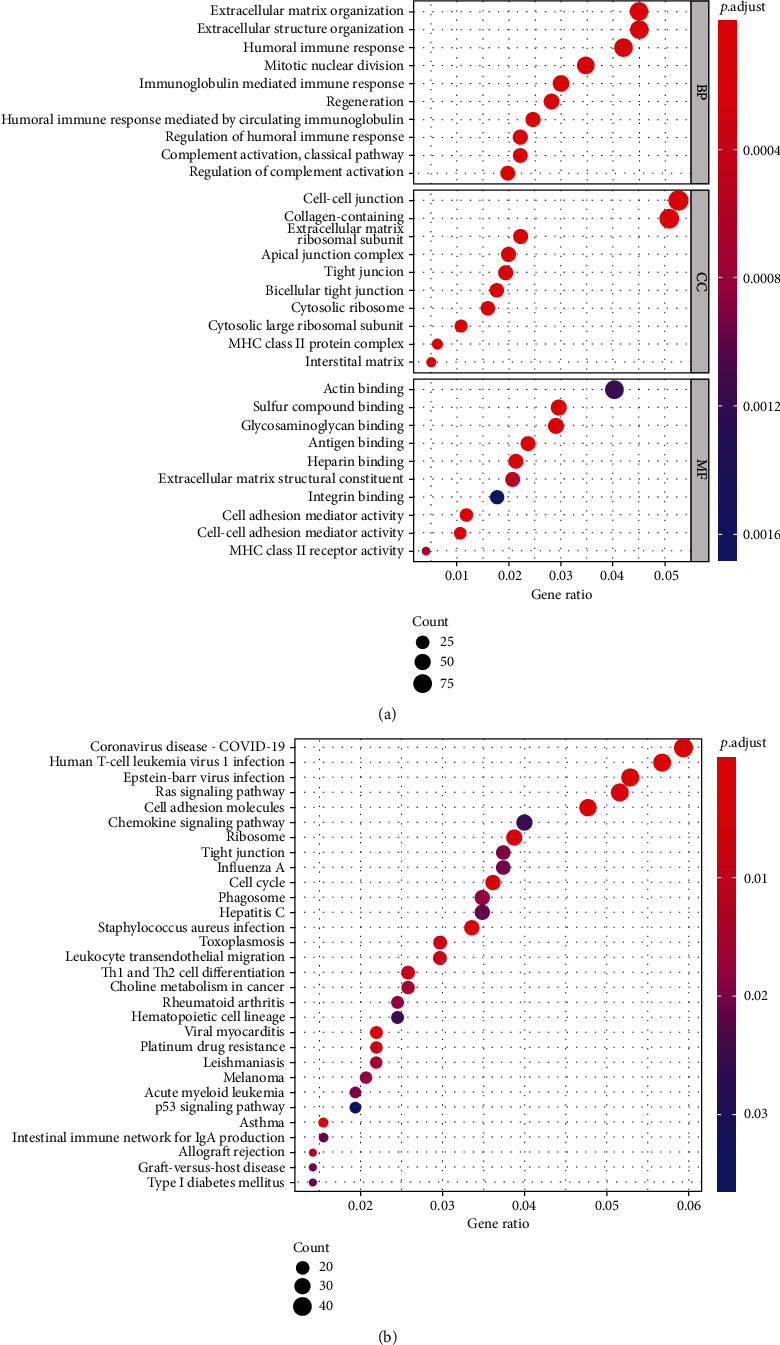
Gene functional enrichment analysis of immune-related DEGs. (a) GO analysis. (b) KEGG pathways.

First, we conducted the GO analysis on 84 immunr-related DEGs. As shown in Figure 3(a), the results were presented in three aspects: (1) biological processes (BP); (2) cellular components (CC); and (3) molecular functions (MF). For BP, DEGs were mainly enriched in the humoral immune response, extracellular matrix organization, and extracellular structure organization. About CC, DEGs were primarily clustered in the cell−cell junction, collagen−containing, and extracellular matrix. Concerning MF, DEGs were mainly associated with actin binding, sulfur compound binding, and glycosaminoglycan binding. Subsequently, the KEGG pathway enrichment analysis was conducted (Figure 3(b)). Their participation in the Human T−cell leukemia virus 1 infection, or in the Epstein−Barr virus infection also suggested that these genes were critical in the inflammatory response.

### 3.3. Construction of the Immune-Related Prognostic Signature

**Figure 4:**
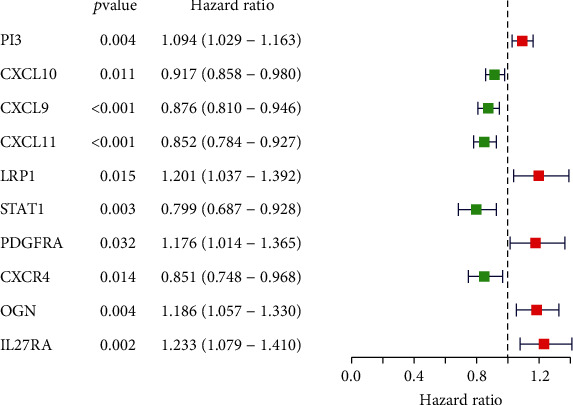
Forest plot of 10 prognostic immune-related DEGs in OC. Hazard ratios (HR) and corresponding 95% confidence intervals (CI) were calculated by the univariate Cox regression model. The red modules are on behalf of the high-risk genes, and the green modules mean the low-risk genes in OC.

We utilized the TCGA-OC dataset (*n* = 346) as a training cohort for the prognostic formulation construction. Univariate Cox regression analysis was conducted to figure out 10 IRPGs which were related with OS at a 0.05 significance threshold (Figure 4), among which PI3, LRP1, PDGFRA, OGN, and IL27RA were the risk factors for OC with HRs of >1, while CXCL10, CXCL9, CXCL11, STAT1, and CXCR4 were the protective factors for OC with HRs of <1. We also investigated the protein expression level of some of these genes by immunohistochemical staining in normal and ovarian cancer tissues by searching in the HPA database (Supplementary Figure 1>).

These 10 IRPGs were later subjected to lasso cox analysis, and 5 IRPGs were finally chosen to be included in the risk model. And their coef was calculated in this way (Table 1). The specific risk-value calculation formula is as follows: Risk score = 0.104∗Exp of PI3 − 0.058∗Exp of CXCL9 − 0.161∗Exp of CXCL11 + 0.164∗Exp of OGN + 0.252∗Exp IL27RA.

Based on the risk score estimated, the patients were separated into high- or low-risk groups based on the median value.

### 3.4. TF Regulatory Network

**Figure 5:**
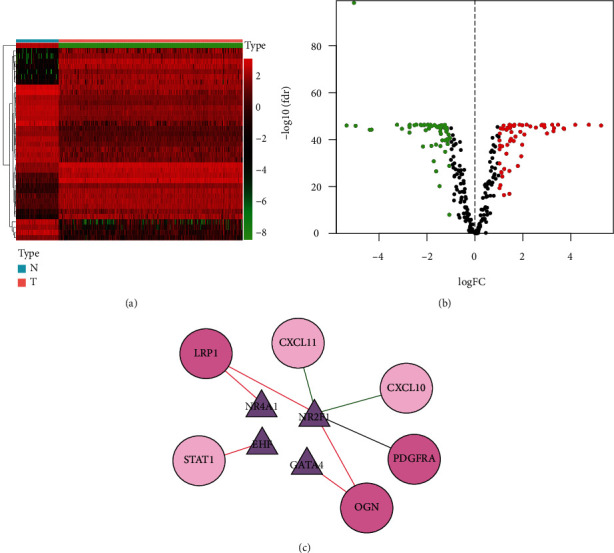
Identification of differentially expressed TFs for ovarian cancer. Heatmap (a) and a volcano plot (b) show distinctly expressed TFs between ovarian cancer tissues and normal tissues. The color in the heatmap from green to red represents the progression from low expression to high. The areas below the blue band represent normal samples, and the areas below the red band represent tumor samples. Colored dots in the volcano map represent differentially expressed TFs. Red on behalf of higher expression while green means lower expression in OC.(c) 4 important TFs involved in immune regulation and prognosis of OC: Transcription factors are represented by triangles; high-risk immune genes are represented by deep pink; low-risk immune genes are represented by pale pink; the positive regulation relationship is denoted by red connection. The negative control relationship is connected with the green line.

After comparing DEG with TFs from the Cistrome database, we found that 38 TFs were diversely expressed in OC and normal ovarian tissues (log FC| >1, *P* *value* < 0.05), including 18 overexpressing and 20 downexpressing TFs. The results are as demonstrated in the volcano plot and heatmap (Figures 5(a) and 5(b)). By performing univariate Cox analysis, 4 prognosis TFs were filtered. Subsequently, we conducted the correlation analysis to further investigate the regulatory relationship between 4 TF and 10 IRPGs (correlation coefficient >0.3, *P* < 0.05). After obtaining the correlation and node attribute table, we used the Cytoscape software to get the TF and immune gene regulatory network diagram.

As shown in Figure 5(c), there were mainly four transcription factors, among which NR2F1 could positively regulate high-risk immune genes LRP1, PDGFRA, and OGN and negatively regulated with two low-risk immune genes, CXCL10 and CXCL11. The other three TF genes, NR4A1, EHF, and GATA4, were involved in the positive regulation of LRP1, STAT1, and OGN, respectively.

### 3.5. Evaluation in the Training Cohort

**Figure 6:**
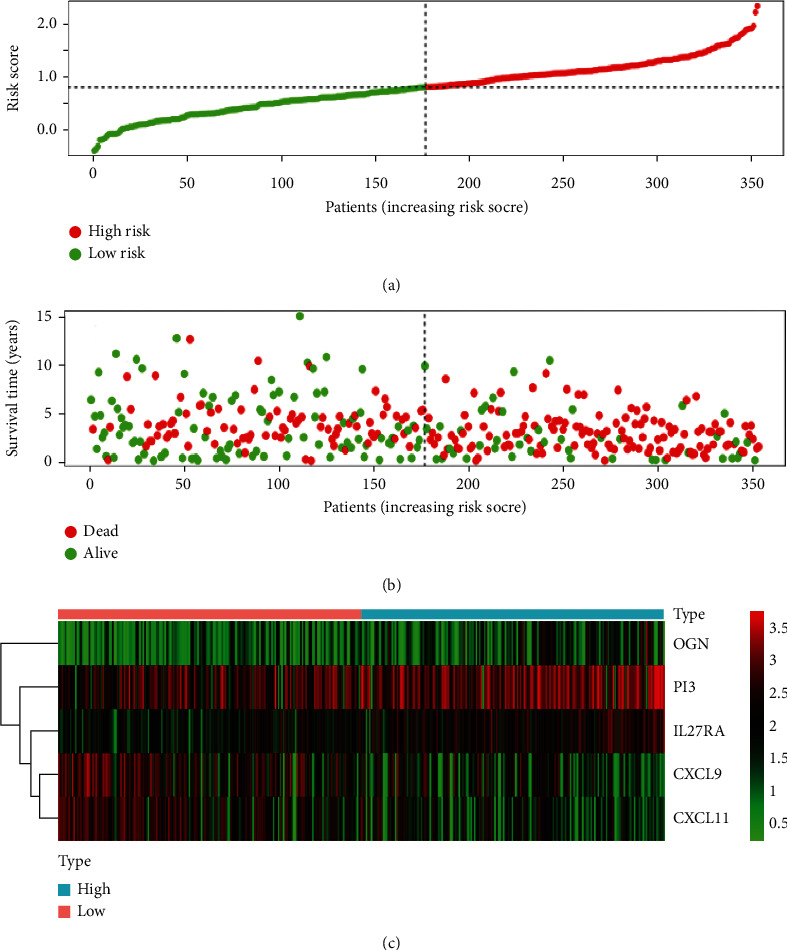
Establish the immune-related prognostic model for OC in the TCGA database. (a) The risk score distribution of OC patients in the TCGA database. The progression from low- to high-risk scores is represented by the color from green to red. (b) Survival status and survival time of each OC patient. (c) Heatmap of the expression of 5 prognostic immune-related genes in OC patients. The color in the heatmap from green to red refers to the progression from low expression to high expression. The areas below the blue band stand for patients with high-risk scores, and the areas below the red band stand for patients with low-risk scores.

**Figure 7:**
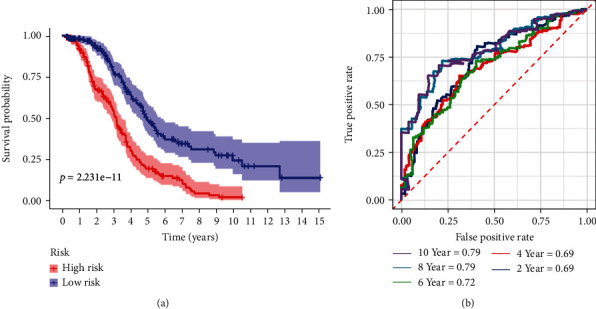
(a) Survival analysis and comparison of OS rate between the low-risk and high-risk groups. (b) Time-dependent ROC curves of OS for the prognostic model.

According to their risk score, TCGA patients were separated into high and low-risk groups (Figure 6(a)). Subsequently, a survival status overview and 5 prognosis genes expression heatmap were drawn (Figures 6(b) and 6(c)). The survival curve was then conducted by K-M survival analysis in the training cohort, which confirmed that the patients with high-risk scores had a significantly shorter OS than those with low scores (*P* < 0.001, Figure 7(a)). Time-dependent ROC curve was also depicted to show the prognostic accuracy, which showed that the AUCs at 2, 4, 6, and 8 years were 0.69, 0.69, 0.72, 0.79, and 0.79, respectively (Figure 7(b)), proving that the prognosis model possessed high sensitivity and specificity, especially in proposing long-term survival probability.

### 3.6. Validation with the Validation Dataset

**Figure 8:**
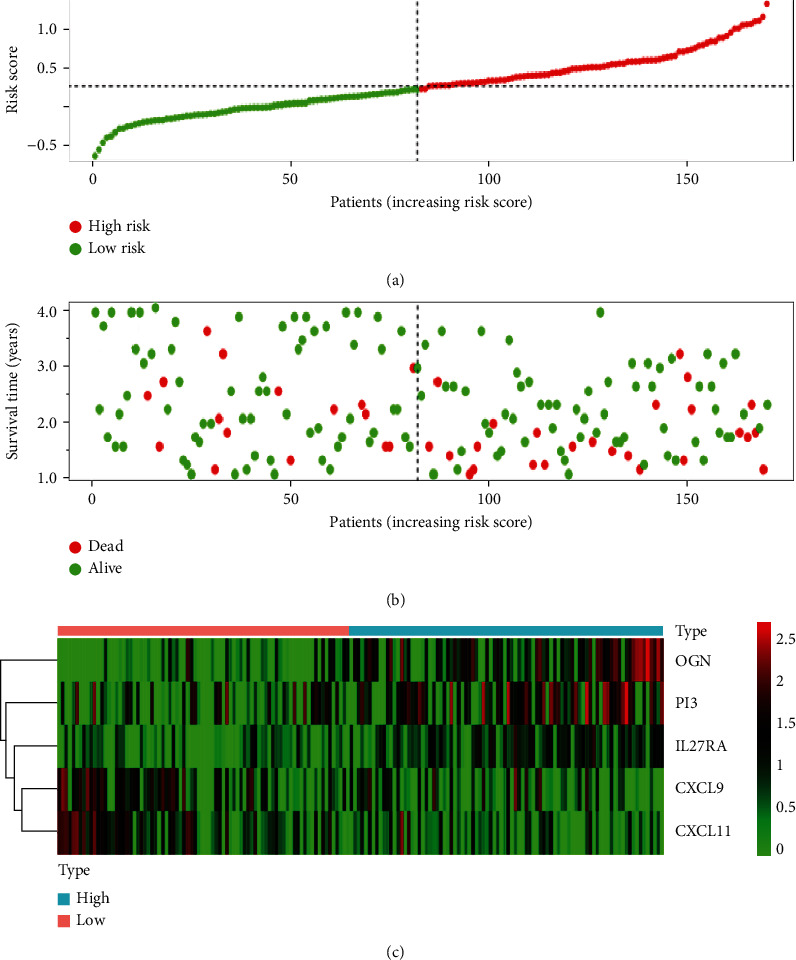
Validation of the immune-related prognostic model for OC in GEO database. (a) The risk score distribution of OC patients in the GEO database. The transition from low-to high-risk scores is presented by the color from green to red. (b) Survival status and survival time of each OC patient. (c) Heatmap of the expression of 5 prognostic immune-related genes in OC patients. The color in the heatmap from green to red represents the progression from low expression to high expression. The areas below the blue band on behalf of patients with high risk and the areas below the red band on behalf of patients with low risk.

**Figure 9:**
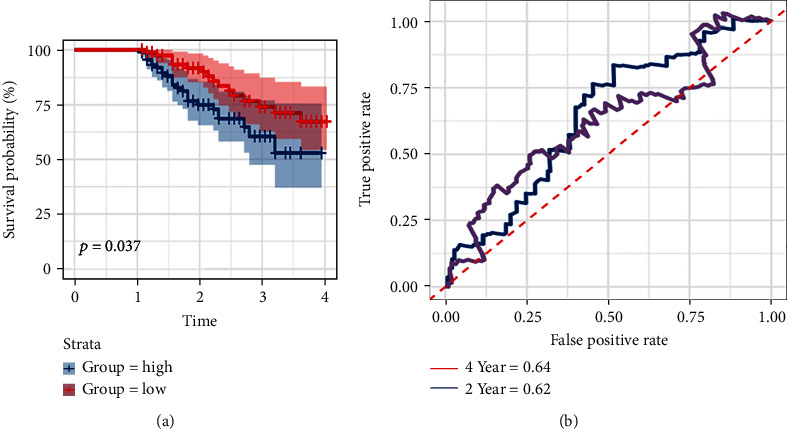
(a) Survival analysis of patients with OS >1 year in the validation dataset. (b) Time-dependent ROC curves of OS for the prognostic model.

The GEO dataset was utilized for further validation analysis to reconfirm the immune prognostic model's reliability. As same as we did in the previous steps, we divided patients into high-risk and low-risk sets. The distribution of risk scores and survival status for each patient in different groups of the validation dataset demonstrated consistent results to the training cohort (Figure 8). Although *P* values in GEO's comprehensive validation database (*n* = 178) were not satisfactory enough (*P* = 0.15, Supplementary Figure 2), we found that it was still of fair value for predicting the prognosis of people with OS >1 year (*n* = 170, *P* = 0.037, Figure 9(a)). Consistent with the training dataset results, AUC reached 0.62 and 0.64 at 2 and 4 years, respectively, which affirm the stability of the model again (Figure 9(b)).

### 3.7. Verification of the Independent Prognostic Value of the Risk Model

**Figure 10:**
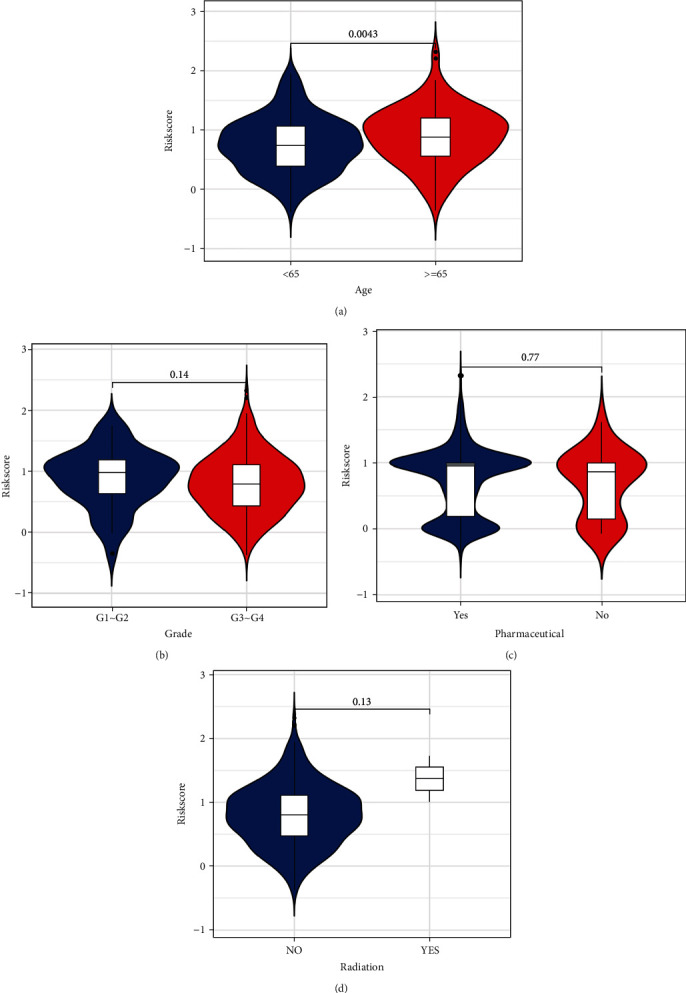
Association between clinical features: age (a), grade (b), pharmaceutical (c), radiation (d), and risk score based on the immune-related prognostic signature.

Given the importance of clinical application, we examined the association between the risk value and clinical parameters in TCGA. However, a significant correlation was only detected between risk scores and age, but not grade, pharmaceutical, or radiation (Figure 10).

**Figure 11:**
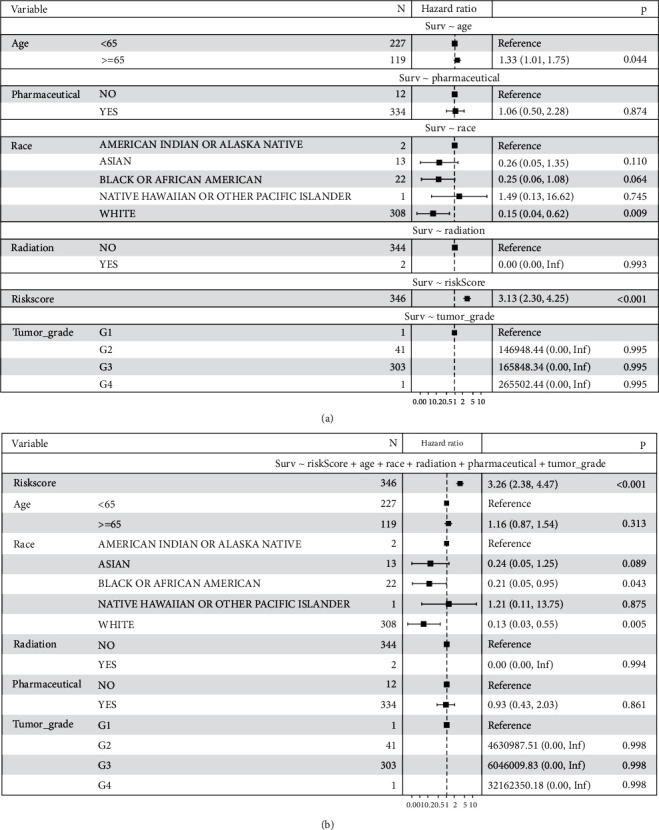
Univariate (a) and multivariate (b) analyses of prognostic factors in the TCGA training cohort identified the signature as an independent prognostic factor.

**Figure 12:**
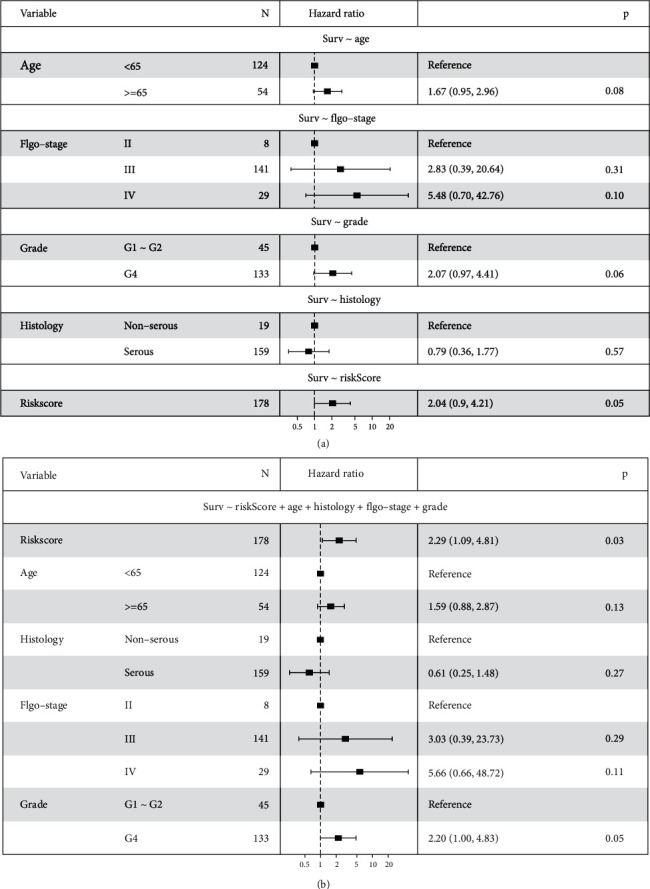
Univariate (a) and multivariate (b) analyses of prognostic factors in the GEO validation cohort identified the signature as an independent prognostic factor.

Subsequently, we conducted univariate and multivariate Cox regression tests to prove the independent prognostic value of the model with different clinical parameters, including age, stage, pharmaceutical, race, radiation, grade, and histology. The statistical summary results are demonstrated in forest plots. The univariate analysis revealed that the high-risk score, the aged, and white people were high-risk factors (*P* < 0.05). Subsequently, the multivariate analyses further confirmed the risk score, age, and race as independent prognostic factors (*P* < 0.001) (Figure 11). To verify its stability, we performed a secondary validation with GEO. Univariate and multivariate Cox regression analysis confirmed that the risk score was still an independent stimulate for prognosis (univariate: *P* = 0.05; multivariate: *P* = 0.03) (Figure 12). These outcomes further confirmed that the 5 IRPGs still have a prognostic value under the influence of other clinical parameters.

### 3.8. Evaluation of Infiltrating Immune Cells of OC

**Figure 13:**
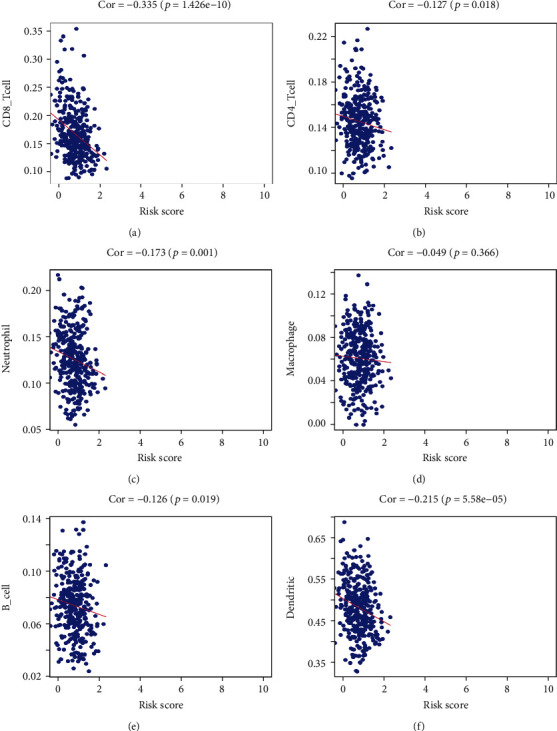
Correlation between the prognostic signature and immune cells including CD8+ T cells (a), CD4+ T cells (b), neutrophils (c), macrophages (d), B cells (e), and dendritic cells (f).

Infiltration of immune cells in TME is an indispensable step both in the initiation and progression of the tumor, and they have been used as biomarkers for immunotherapeutic response [16]. In this step, we investigated the correlation between the signature with the content of 6 types of immune cells from the TIMER database. It revealed that B cells (*P* = 0.019), dendritic cells (*P* = 5.58*e* − 05), CD8+ T cells (*P* = 1.426*e* − 10), CD4+ T cells (*P* = 0.018), and neutrophils (*P* = 0.001) had a prognostic value for OC patients, and as the curves depicted, they were all negatively correlated (Cor <0). The above results confirmed that the prognostic model could inflect the infiltrating immune cells in the tumor microenvironment (Figure 13).

## 4. Discussion

OC remains a headache for physicians and females over the world due to its high rate of recurrence and resistance caused by hidden precursors. Given the importance of TME in OC, this study aims to identify IRGs with predictive value and to establish a more accurate prognostic model. First, transcriptome and clinical data of OC and normal ovarian tissue were downloaded from the TCGA database and GTEx database, respectively. After integration, the obtained 84 immune-related DEGs were verified to participate in multiple immune-related pathways by the KEGG and GO analysis. Subsequently, univariate Cox regression analysis was conducted to determine prognostic-related DEGs. A total of 10 IRGs were identified as IRPGs. Subsequently, we constructed a TF-mediated network to explore its internal mechanism. Then, lasso regression analysis was used to construct a vigorous risk model based on 5 IRPGs and their coef value, which can mirror prognosis of patients with OC , in particular, long-term survival. Compared with other clinical factors, we demonstrated its superior predictive ability. Due to GSE49997 containing sufficient tumor samples with completed clinical information, it was chosen to validate its reliability, and the model has proven to withstand external databases. In addition, we found that the model can also well reflect the tumor immune microenvironment by exploring it in the TIMER database.

Cancer immunotherapy has become the focus of cancer research because of its unique long-term responses in refractory patients. Nevertheless, the immunotherapy response rates in OC remain low [17]. At present, two factors, TILs and PD-1 ligand (PD-L1), were recognized as biomarkers to preview patients' response to immune checkpoint inhibitors (ICIs) [18]. Given this, more than half of high-grade serous ovarian cancers were proposed to exhibit resistance to immune therapy. In comparison, other histological types show low resistance [19]. Several ICIs have been used in early clinical trials of OC therapy in combination, for example, anti-PD-1 antibodies including pembrolizumab, nivolumab, durvalumab, and avelumab, and also antibodies targeting CTLA-4 including ipilimumab and atezolizumab [20, 21]. However, preliminary clinical data suggest that these drugs have limited efficacy against OC, with an objective response percentage of 10-15% [21]. Thus, it remains to be figured out why the therapeutic response of ICIs is not satisfactory. We also need to find high-efficiency biomarkers and determine the best combination of strategies. While early screenings are beneficial for cancer detection and treatment, most of the patients are not willing to cooperate [22]. The patients prefer reliable prognostic models to guard their health situation. Thanks to the easy access to the well-developed databases of genetic and clinical information, statistical predictive models have been improved for various malignancies, for instance, colorectal cancer, hepatocellular carcinoma, gastric cancer, and esophageal cancer [23–26]).

Our study still has some drawbacks. Although we have the advantage of using multiple databases to construct and validate the risk model, it is still characterized by a retrospective design. Therefore, prospective cohort studies are needed. What's more, the association between IRGs and clinical parameters was not strongly sufficient. But fortunately, multivariate Cox analysis has demonstrated its independent prognostic value after adjustment for other factors. Given this, for the clinical application of this model, we still need more databases and clinical data, and we should better combine them with clinical features for more individualized combination therapy. Moreover, we also welcome exploring the applicability of our model to other female reproductive diseases, such as cervical cancer, endometrial carcinoma, and pelvic inflammation disease.

In summary, we have constructed a prognosis model consisting of 5 IRPGs for OC, which may also serve as a novel reference parameter for identifying patients who will benefit from immunotherapy.

## Figures and Tables

**Table 1:** Risk genes' coef in the prognostic risk model.

Gene	Coef
PI3	0.104421899319749
CXCL9	-0.0584230498809408
CXCL11	-0.0160551892127128
OGN	0.164347646094798
IL27RA	0.252033958859281

## Data Availability

The data that support the findings of this study are openly available in TCGA (https://portal.gdc.cancer.gov) GEO (https://www.ncbi.nlm.nih.gov/geo/query/acc.cgi?acc=GSE49997), GTEx database (https://commonfund.nih.gov/GTEx), TIMER database (https://cistrome.shinyapps.io/timer/), and ImmPort (http://www.immport.org).

## Additional Points

*Impact statement*. The immune-related genes (IRGs) have demonstrated their indispensable importance in the occurrence and progression of OC. Therefore, this study aimed to figure out IRGs with predictive value and build a prognostic model for a more accurate assessment, as well as help present novel ideas for targeted therapy for OC.

## Supplementary Materials

Supplementary Materials Supplementary Figure 1. Immunohistochemical staining of IRPGs in normal and ovarian cancer tissues. Supplementary Figure 2. Survival analysis between the low-risk and high-risk groups in the total validation cohort.
