# Supplementary figures and images for: Systematic Construction and Validation of an Immune-Related Gene-Based Model to Predict Prognosis for Ovarian Cancer
Source: Biomed Res Int. 2022 Apr 21;2022:7356992. doi: 10.1155/2022/7356992 (PMC9050317; doi:10.1155/2022/7356992)

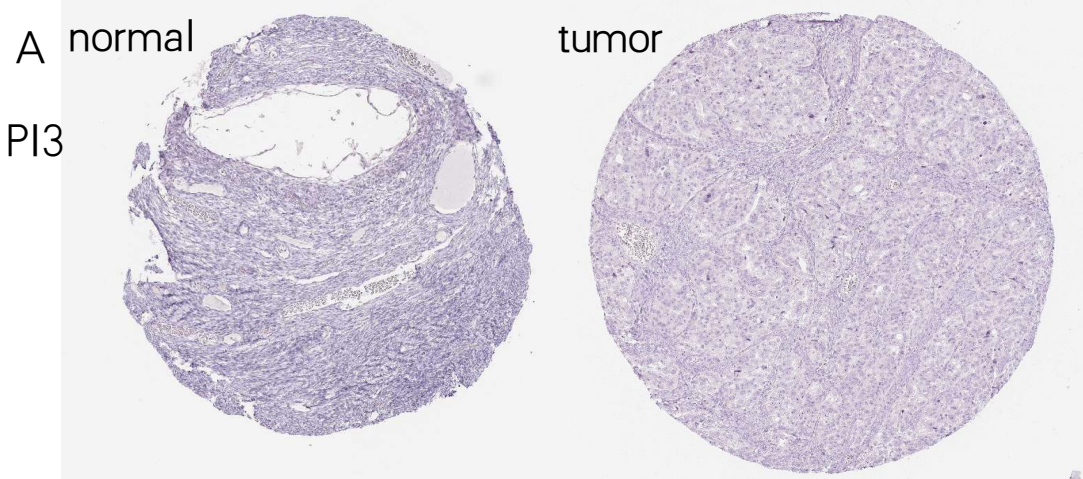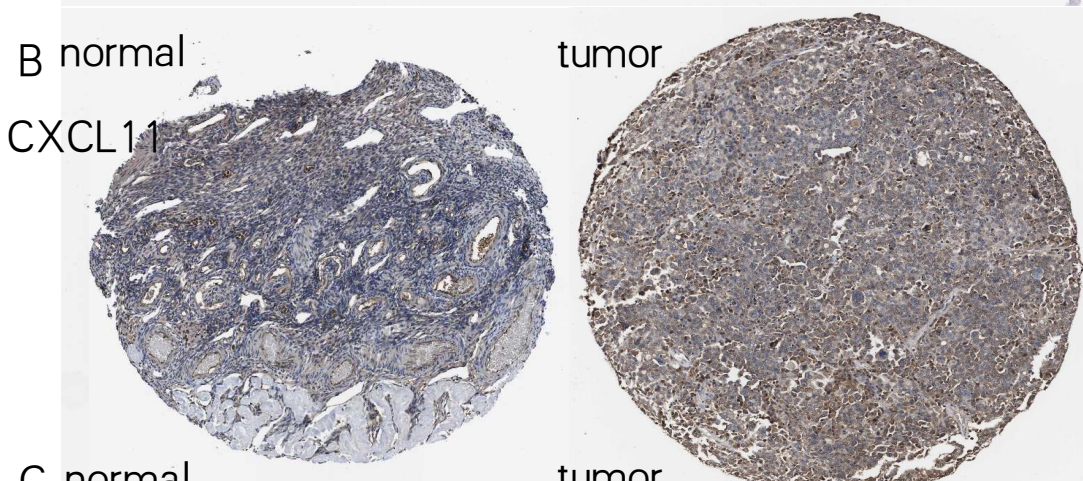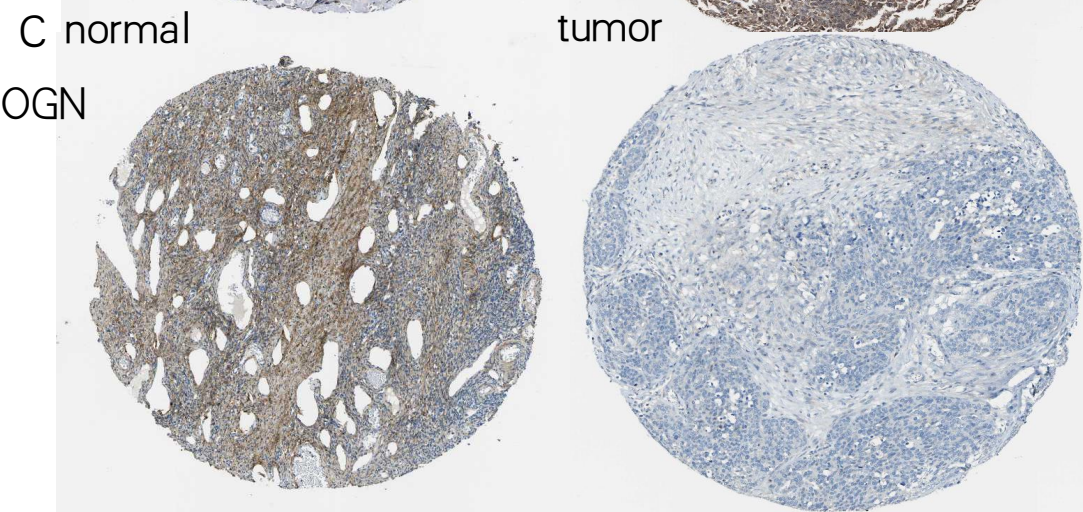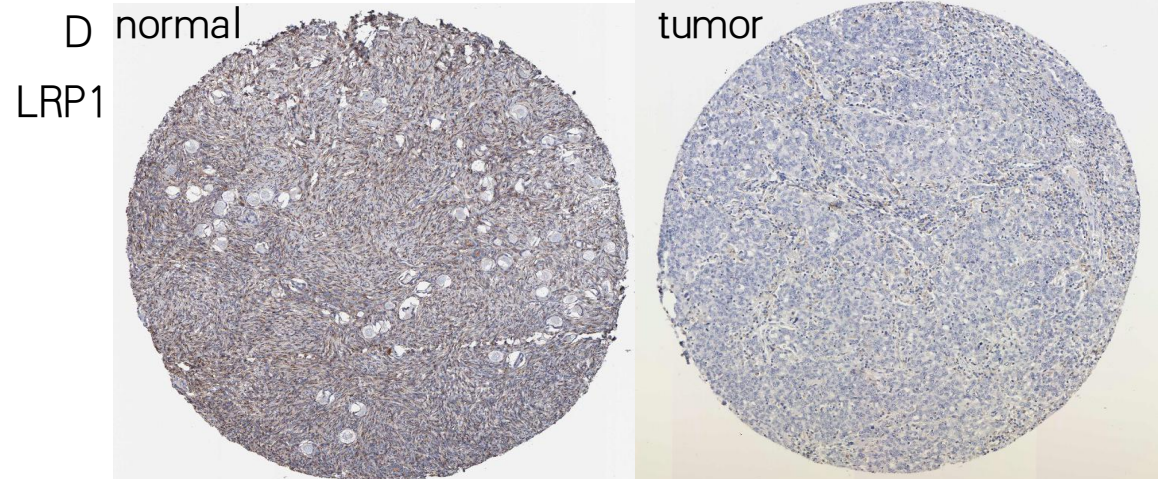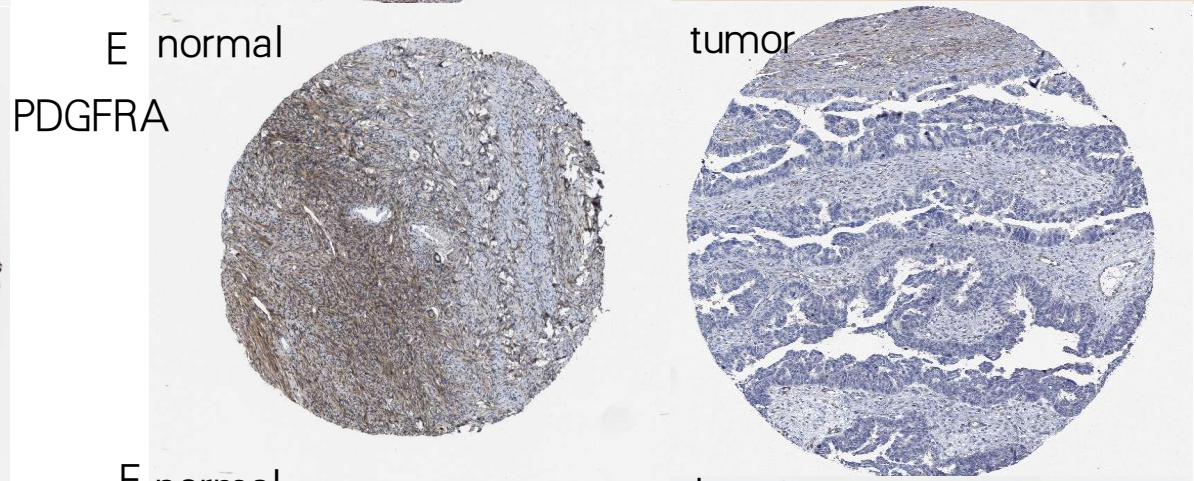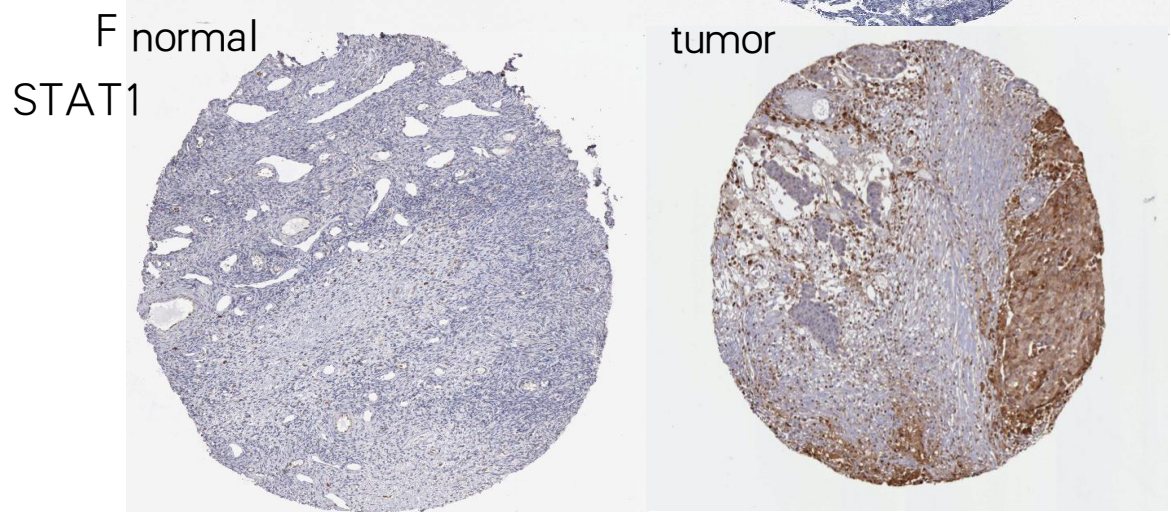

Supplement: Supplementary Materials — Supplementary Figure 1. Immunohistochemical staining of IRPGs in normal and ovarian cancer tissues. Supplementary Figure 2. Survival analysis between the low-risk and high-risk groups in the total validation cohort. [file 7356992.f1.zip › supplementary figure1.pdf]

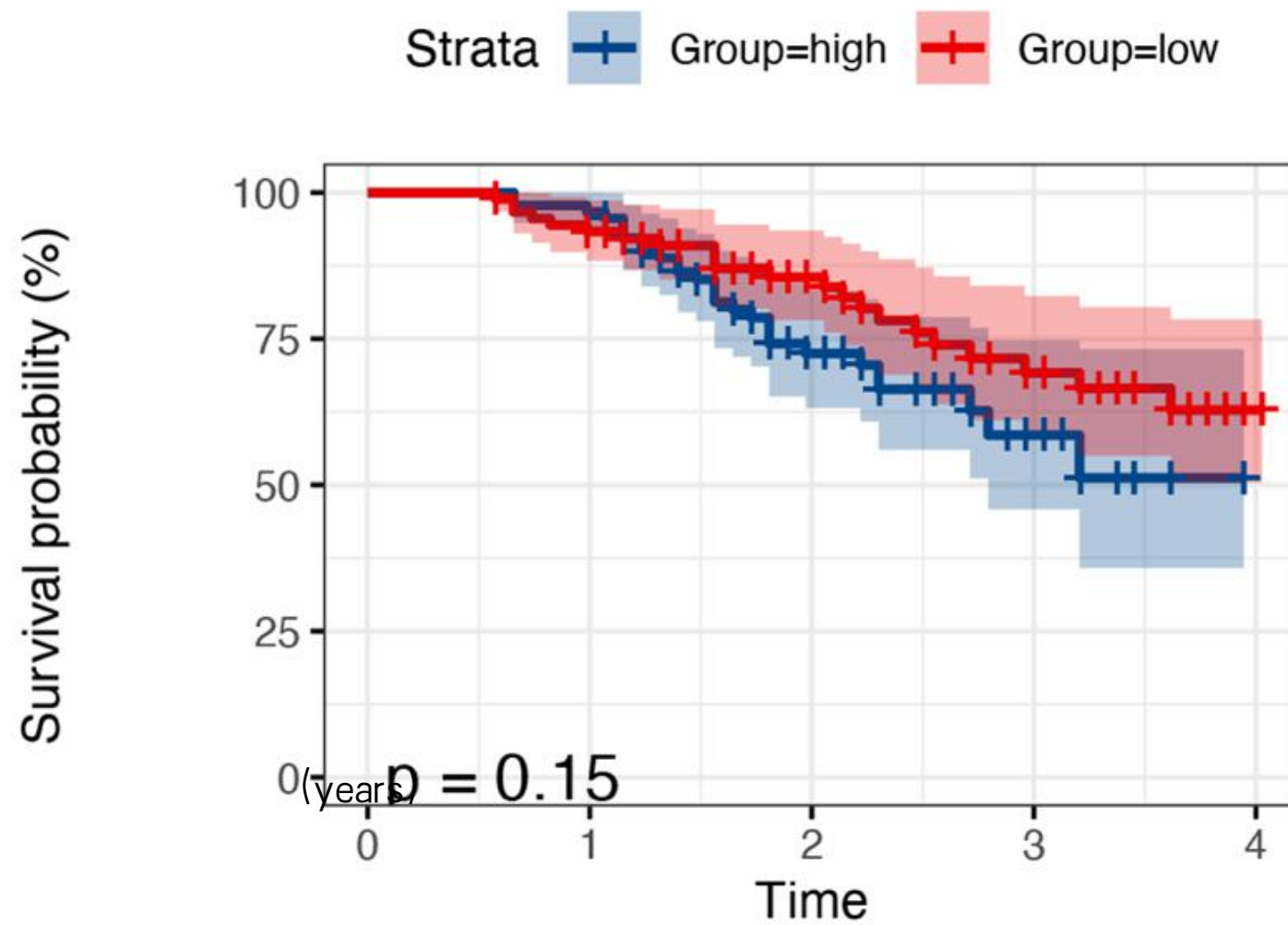

Supplement: Supplementary Materials — Supplementary Figure 1. Immunohistochemical staining of IRPGs in normal and ovarian cancer tissues. Supplementary Figure 2. Survival analysis between the low-risk and high-risk groups in the total validation cohort. [file 7356992.f1.zip › supplementary figure2.pdf]
